# Supplementary figures and images for: Higher Preoperative Maximum Standardised Uptake Values (SUVmax) Are Associated with Higher Biochemical Recurrence Rates after Robot-Assisted Radical Prostatectomy for [68Ga]Ga-PSMA-11 and [18F]DCFPyL Positron Emission Tomography/Computed Tomography
Source: Diagnostics (Basel). 2023 Jul 11;13(14):2343. doi: 10.3390/diagnostics13142343 (PMC10378114; doi:10.3390/diagnostics13142343)

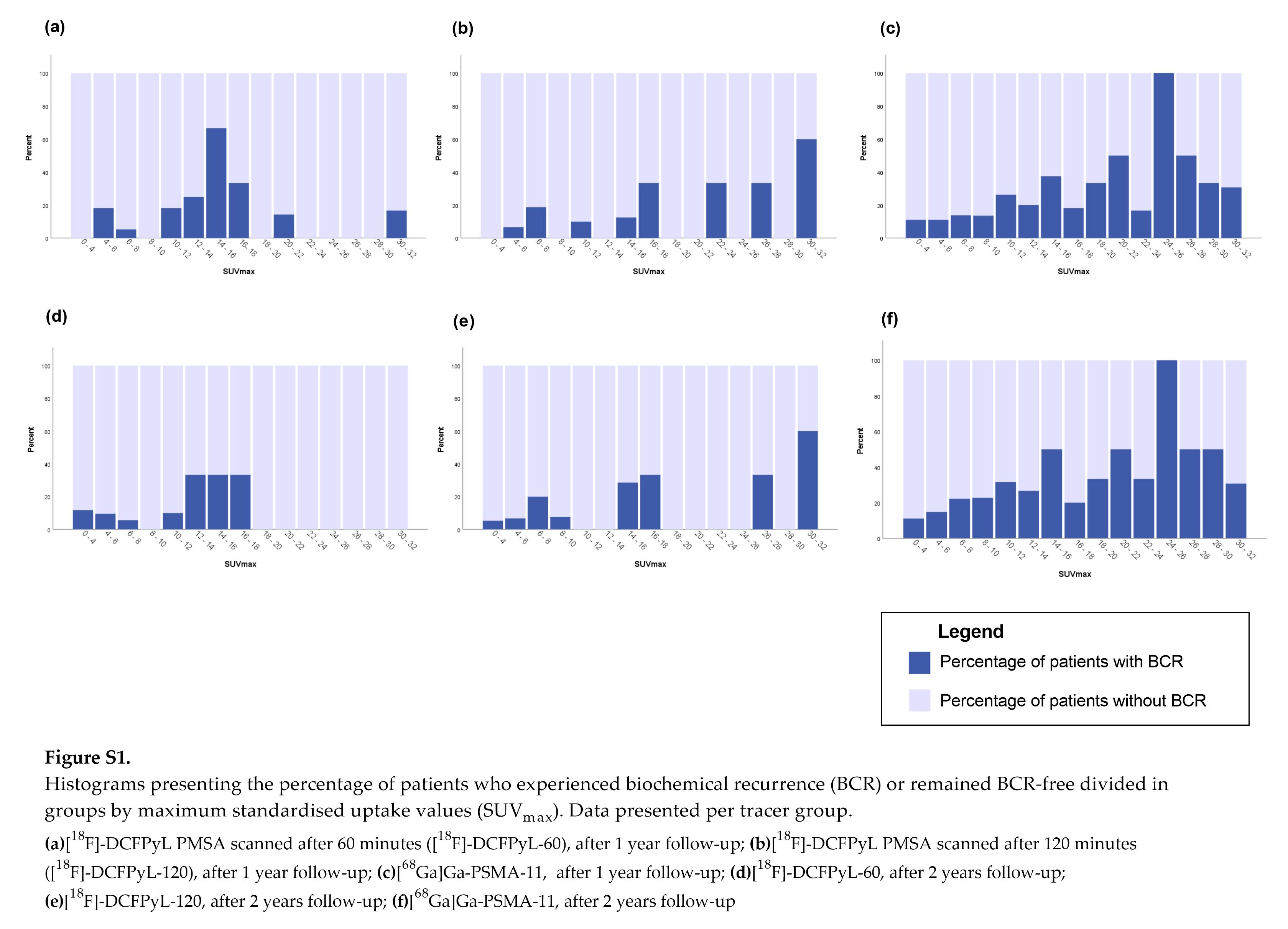

Supplement: Supplementary file 1 [file diagnostics-13-02343-s001.zip › diagnostics-2451935-supplementary.jpg]
